# Disruption to test scores after hurricanes in the United States

**DOI:** 10.1088/2752-5309/adb32b

**Published:** 2025-02-14

**Authors:** Gabriella Y Meltzer, G Brooke Anderson, Xicheng Xie, Joan A Casey, Joel Schwartz, Michelle L Bell, Yoshira Ornelas Van Horne, Jared Fox, Marianthi-Anna Kioumourtzoglou, Robbie M Parks

**Affiliations:** 1 Department of Obstetrics and Gynecology, Columbia University Irving Medical Center, New York, NY, United States of America; 2 Department of Environmental and Radiological Health Sciences, Colorado State University, Fort Collins, CO, United States of America; 3 Department of Biostatistics and Informatics, Feinberg School of Medicine, Northwestern University, Chicago, IL, United States of America; 4 Department of Environmental and Occupational Health Sciences, University of Washington School of Public Health, Seattle, WA, United States of America; 5 Department of Environmental Health Sciences, Mailman School of Public Health, Columbia University, New York, NY, United States of America; 6 Department of Environmental Health, Harvard T.H. Chan School of Public Health, Boston, MA, United States of America; 7 Department of Environmental Health, Yale University School of the Environment, New Haven, CT, United States of America; 8 Fox EduConsulting, Chevy Chase, MD, United States of America; 9 Environmental Health Sciences, University of California Los Angeles, Los Angeles, CA, United States of America

**Keywords:** tropical cyclones, hurricanes, educational attainment, standardized test scores

## Abstract

Quantifying how hurricanes disrupt educational attainment is essential to evaluating the burden of climate-related disasters. Here, we examine the association between hurricane-force tropical cyclones and educational attainment among elementary and middle school students in all affected areas in the United States during the 2008/2009–2017/2018 school years. Educational performance was based on county-level average standardized test scores in math and reading/language arts (RLAs). Hurricane-force tropical cyclone-exposed counties were those that experienced a sustained maximal wind speed ⩾64 knots. We estimated the association between hurricane-force tropical cyclone exposure and long-term test scores using a Bayesian hierarchical linear model, accounting for time-varying covariates at the county and grade cohort level. For hurricane-exposed counties, compared with the rest of the state, there were better test scores in Florida in math (*β* = 0.14; 95% CrI: 0.02, 0.26; PP[*β* > 0] = 99.0%) and RLA (*β* = 0.11; 95% CrI: 0.02, 0.22; PP[*β* > 0] = 99.2%), and worse math scores in North Carolina (*β* = −0.16; 95% CrI: −0.29, −0.03; PP[*β* < 0] = 99.4%). Grade cohorts with more racialized and minoritized (e.g. Black, Hispanic, Indigenous) and socioeconomically disadvantaged students tended to have lower test scores, while grade cohorts with greater shares of students racialized as Asian and counties with more college-educated adults tended to have higher scores regardless of hurricane exposure. Disaster preparedness must maximize resilience to climate-related stressors’ impacts on academic achievement, especially for vulnerable populations.

## 1. Introduction

Tropical cyclones, such as hurricanes and tropical storms, are intense circular storms that originate over warm tropical oceans and are characterized by low atmospheric pressure and high windspeeds. They draw energy from the sea surface and maintain strength as long as they remain over warm water (Zehnder 2023). Hurricanes are very active in the United States; the 2020 Atlantic hurricane season was the most active on record (Blackwell 2020), and 2021 was the third time that the storm naming system was exhausted (Harvey 2021). Hurricanes will continue to pose a threat to the United States as they make longer landfall and peak closer to land than in previous years (Chavas and Chen 2020, Wang and Toumi 2021). Once hurricanes make landfall, they can be extremely disruptive and very destructive. From 1900 to 2017, hurricanes inflicted $2 trillion in damages, equating to $17 billion annually in the United States (Weinkle *et al* 2018).

While there is evidence that hurricanes are associated with deaths (Parks *et al* 2023, 2022) and hospitalizations (Parks *et al* 2021) from many major causes, less is known about their societal burden on medium and long-term mental and behavioral health, including educational attainment (Parks and Guinto 2022, Grineski *et al* 2024). Academic achievement during childhood and adolescence has been shown to shown to be strongly associated with concurrent health risk behaviors including violence, tobacco use, alcohol and drug use, unsafe sex practice, sedentariness, and poor eating (Bradley and Greene 2013, Skalamera and Hummer 2016). Educational attainment is also a strong predictor of mental health (Jareebi and Alqassim 2024), self-rated health (Lê-Scherban *et al* 2014, Elovainio *et al* 2016), risk of chronic disease (Choi *et al* 2011, Magnani *et al* 2024), and overall mortality and life expectancy (Kaplan *et al* 2014, Puka *et al* 2022). Children and adolescents, who are particularly susceptible to climate-related disasters (Peek *et al* 2018), will experience more frequent and severe hurricanes in their lifetimes than previous generations due to climate change (Thiery *et al* 2021). Hurricanes that destroy school buildings and displace students and teachers may cause children to miss school, have poorer academic performance and delayed progress, or fail to complete their education altogether (Peek 2008). Hurricane Katrina in 2005, for example, displaced 348 000 students across Louisiana, Mississippi, and Alabama (Picou and Marshall 2007) and destroyed nearly 80% of New Orleans’s public school buildings (Klein 2015). The strongest tropical cyclones (hurricane-force winds) have had long-lasting deleterious impacts on education systems in highly impacted communities throughout the United States (Davis *et al* 2021).

Several studies, most of which examined the aftermath of Hurricanes Katrina and Rita, have identified the adverse effects of individual major hurricanes on student educational outcomes such as academic achievement, negative behaviors, and school attendance (Holmes 2002, Ward *et al* 2008, Weems *et al* 2013, Scott *et al* 2014, Lai *et al* 2019). Despite this research, no study to date has comprehensively assessed the impact of hurricanes on educational attainment over multiple years of study across the entire United States or assessed differences across climatically and politically differing states, which also have varying capabilities in disaster preparedness, response, and recovery. Here, we examined the association between hurricane-force tropical cyclones and educational attainment among elementary- and middle school-age students in all affected counties of the United States. Our objectives were to (1) estimate the association between hurricane-force tropical cyclone exposure and long-term effects on math and reading/language arts (RLAs) test scores in United States counties and (2) to evaluate how these effects vary by state.

## 2. Methods

### 2.1. Outcomes

We ascertained educational attainment based on annual standardized test scores in math and RLA administered in the spring to public school third to eighth grade students across 2420 counties in the contiguous United States as mandated by the No Child Left Behind Act of 2001 (Boehner 2002). We retrieved average test score data aggregated at the county level from the Stanford Education Data Archive (SEDA), which were available for academic years 2008–2009 to 2017–2018 (Reardon *et al* 2022). We only included states if they contained at least one county that experienced at least one hurricane during our study period. SEDA data adjusted for interstate differences in academic proficiency using the National Assessment of Educational Progress (NAEP), an annual exam administered at the same time on the same academic content to a representative sample of United States students (Sharp 2019). The SEDA test scores are centered at the grade level and scaled such that a score of 4, for example, is equal to the average national NAEP score across four cohorts of students in fourth grade in the spring of 2009, 2011, 2013, and 2015. According to SEDA documentation, ‘1 unit in this metric is equal to the average per-grade increase in scores between fourth and eighth grade for those same cohorts, assuming usual grade promotion.’ This allows scores to be comparable across the entire United States, over time, and across grades (Reardon *et al* 2022).

### 2.2. Exposure

We obtained data on tropical cyclone wind exposure in the United States with full space and time coverage over the study period of 2008–2018 from publicly available datasets generated by Anderson *et al* (Anderson 2017, Anderson and Eddelbuettel 2017, Anderson *et al* 2020). We used daily estimates of maximum wind sustained speed by county to classify whether a county had been exposed to a hurricane in a given year, after which it was considered exposed for the entire study period (Anderson 2017). We defined hurricane exposure by peak sustained winds in a county’s population center associated with a hurricane at the point of closest approach having reached or exceeded 64 knots or 74 miles per hour. Research has shown that high wind speeds during hurricanes are strongly correlated with flooding, high storm surge, and structural damage, especially in coastal areas (Murnane and Elsner 2012, Chavas *et al* 2013, Musinguzi and Akbar 2021). We lagged hurricane exposure to measure whether standardized test scores based on exams administered in March–May of a given academic year were associated with storms that took place during the previous hurricane season of May–September.

### 2.3. Covariates

We retrieved time-varying, annual covariates at both the grade cohort and county level from SEDA that we considered to be potential confounders and/or effect modifiers of the association between hurricane exposure and standardized test performance (Reardon *et al* 2022). A grade cohort is considered all the students in a specific grade level in a given county. At the grade cohort level, covariates included the percentage of students who identified as Black, Hispanic, Asian, and American Indian/Alaska Native; the percentage of students who received free lunch; and the percentage of students who were considered economically disadvantaged. At the county level, covariates included the percentage of students in urban locale schools; percentage of English-language learner students; percentage of special education students; percentage of adult county residents with a college degree; percentage of county residents living in poverty; and percentage of households headed by single mothers.

### 2.4. Statistical analysis

We developed a Bayesian hierarchical linear model (the formulation of which has been previously referred to as a generalized difference-in-differences approach (Lu *et al* 2021)) with two-way fixed and random effects model to assess the association between hurricane-force tropical cyclone exposure and average annual standardized test scores at the county level (Donald and Lang 2007, McElreath 2016). Bayesian inference is advantageous in that it allows for the full distributional estimation of the parameters of interest, as well as borrowing of information across neighboring (e.g. county) units (Gelman *et al* 2015, Parks *et al* 2022). If a given county had been exposed to a hurricane-force tropical cyclone in a particular year, we treated all associated grade cohorts as exposed for the remainder of the study period. The model m*et al*l necessary assumptions and was based on those in other studies examining the effects of environmental exposures on standardized test scores (Lu *et al* 2021, Wen and Burke 2022). The model was the following:

\begin{equation*}{\text{Scor}}{{\text{e}}_{itg}} = { }{\alpha _0} + \left( {\beta + { }{b_s}} \right){\text{Hurrican}}{{\text{e}}_{it}} + {{\Sigma }}\beta {\text{Covariate}}{{\text{s}}_{itg}} + {\text{ Cohor}}{{\text{t}}_{ig}} + {\text{ Yea}}{{\text{r}}_t} + { }{\varepsilon _{itg}}\end{equation*}

where *i* was the county, *t* was the year, and *g* was the grade. Score*_itg_* was the average standardized test score for grade *g* students in state *s*, in county *i*, in year *t*. Hurricane*_it_* was whether a hurricane-force tropical cyclone occurred in a given year *t* and county *i*. Covariates*_itg_* were covariates for grade *g* students in *i* county in a given year *t*. Cohort*_ig_* and Year*_t_* were cohort and year fixed effects. We used random effects for the hurricane term by state and *ϵ_itg_* was the random error.

We used weakly informative priors so that parameter estimation would be driven by the data. All *β* terms were assigned *N*(0,1000) priors. We assigned all random effects to have a prior of the form *N*(0, *σ*). We assigned priors on random effects (i.e. *b_s_*) to have *σ* ∼ logGamma(*θ,δ*) priors with shape *θ* and rate *δ* = 0.001. We based our reported positive and negative associations on point estimates with two-sided 95% credible intervals. We obtained posterior probabilities that parameter estimates were clear of the null through a formal comparative analysis of 1000 draws from the posterior marginal distribution of each effect estimate. The proportion of draws that was greater than 0 represented the probability that an effect estimate was greater than 0 (Gelman *et al* 2015).

We conducted statistical analysis in R version 4.3.1. We fitted all models using integrated nested Laplace approximation (INLA) executed by the R-INLA software (Rue *et al* 2009).

### 2.5. Sensitivity analysis

We conducted sensitivity analyses with a model including random intercepts by state, and also restricted models to counties whose student enrollment was greater than the 5th and lower than the 95th percentiles, as well as counties that only experienced one hurricane over the study period to account for the potentially cumulative effects of repeated hurricane exposures over the study period. We also conducted sensitivity analyses examining potential moderating effects by grade level; grade cohort-level proportion of students racialized as Black, Hispanic, and Indigenous and socioeconomically disadvantaged students; and county-level poverty rates and proportion of special education students. Given that the addition of county-level and grade cohort-level covariates may have attenuated the association between hurricane-force tropical cyclone exposure and standardized test scores (Schisterman *et al* 2009), we also conducted a sensitivity analysis where we removed all covariates from both the main model, as well as that restricted to counties with a single hurricane exposure over the study period.

## 3. Results

### 3.1. Summary statistics

Between 2009 and 2018, national average standardized math scores increased in younger grade cohorts and decreased in older ones, while RLA scores remained relatively stable (table 1). Some states consistently outperformed others throughout the study period. For example, the median average RLA score in 2018 for eighth-grade cohorts in South Carolina was 7.05 compared to 8.49 in New Jersey. The distributions of standardized test scores for each state included in our analysis at baseline in 2009 and the conclusion of the study period in 2018 are shown in table S1. The median national average proportions of grade cohort students receiving free lunch increased from 40.5% in 2009 to 49.0% in 2018, as did the average percentage of grade cohort students considered socioeconomically disadvantaged (2009 median = 51.0%; 2018 median = 57.2%). In addition, the average median percentage of grade cohort students racialized as Hispanic increased over twofold from 3.0% in 2009 to 6.5% in 2018 (table 1). At the county level nationally, the median average proportion of adult residents with a college degree increased from 15.8% in 2009 to 18.1% in 2018 (table 1).

**Table 1.** Educational and demographic characteristics variables in states exposed to hurricanes in the United States in 2009 and 2018.

		Grade	2009 percentiles					2018 percentiles				
Grade-specific standardized test scores			5th	25th	50th	75th	95th	5th	25th	50th	75th	95th
	Mean standardized math score	3	1.24	2.23	2.82	3.28	3.98	1.73	2.54	3.08	3.57	4.29
		4	2.28	3.20	3.74	4.21	4.87	2.24	3.23	3.92	4.45	5.24
		5	3.12	4.20	4.78	5.20	5.97	2.95	4.04	4.72	5.25	6.14
		6	4.25	5.26	5.77	6.34	7.16	3.83	4.94	5.66	6.26	7.36
		7	5.08	6.17	6.84	7.33	8.22	4.47	5.80	6.49	7.20	8.25
		8	6.15	7.28	7.88	8.45	9.48	5.30	6.61	7.39	8.20	9.30
	Mean standardized RLA score	3	0.96	2.08	2.72	3.37	4.37	1.00	2.06	2.70	3.43	4.30
		4	2.04	2.94	3.49	4.11	5.02	1.90	2.99	3.63	4.35	5.16
		5	2.93	3.94	4.54	5.02	5.95	2.87	3.76	4.44	5.13	6.07
		6	4.06	5.00	5.52	6.05	6.89	3.84	4.77	5.35	6.10	6.99
		7	4.90	5.83	6.40	6.88	7.73	4.89	5.77	6.40	6.98	7.91
		8	5.92	6.87	7.39	7.90	8.68	5.98	6.84	7.36	7.95	8.84

Grade cohort level variables	Percent American Indian/Alaska Native		0.00	0.00	0.00	0.01	0.02	0.00	0.00	0.00	0.00	0.02
	Percent Asian		0.00	0.00	0.01	0.01	0.05	0.00	0.00	0.01	0.02	0.06
	Percent Hispanic		0.01	0.04	0.14	0.33	0.77	0.02	0.10	0.24	0.44	0.83
	Percent Black		0.00	0.04	0.14	0.31	0.67	0.00	0.02	0.10	0.26	0.61
	Percent Free Lunch		0.21	0.37	0.45	0.55	0.72	0.26	0.46	0.55	0.68	0.97
	Percent Economically Disadvantaged		0.30	0.48	0.57	0.67	0.84	0.34	0.52	0.61	0.72	0.86

County level variables	Percent English Language Learners		0.00	0.01	0.04	0.08	0.17	0.00	0.02	0.05	0.09	0.17
	Percent Urban Schools		0.00	0.00	0.00	0.00	0.58	0.00	0.00	0.00	0.00	0.58
	Percent with College Degree		0.09	0.12	0.16	0.21	0.34	0.10	0.14	0.18	0.24	0.40
	Percent Living in Poverty		0.09	0.13	0.17	0.20	0.27	0.09	0.13	0.16	0.20	0.26
	Percent Single-Mother Households		0.11	0.15	0.18	0.22	0.27	0.12	0.15	0.18	0.22	0.29
	Percent Special Education		0.08	0.11	0.12	0.14	0.18	0.08	0.10	0.12	0.14	0.18

**Figure 1.**
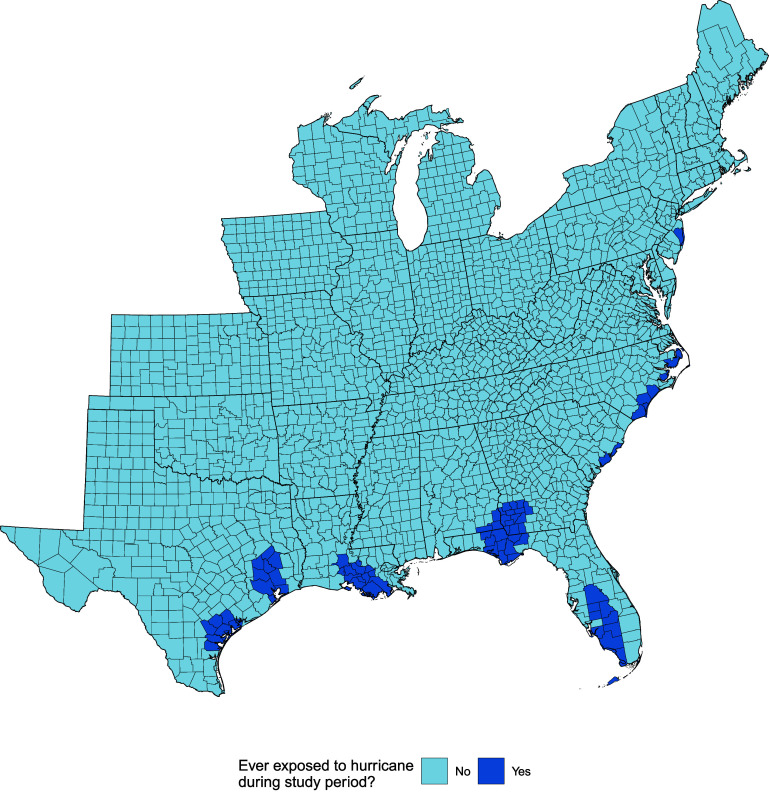
Counties exposed to hurricane-force tropical cyclones during 2009–2018.

There were 74 counties exposed to hurricane-force tropical cyclones over the course of the study period (figure 1).

In the figure above, counties highlighted in dark blue are those that were exposed to hurricane-force tropical cyclones over the course of the study period.

### 3.2. Association of hurricanes with math scores

**Figure 2.**
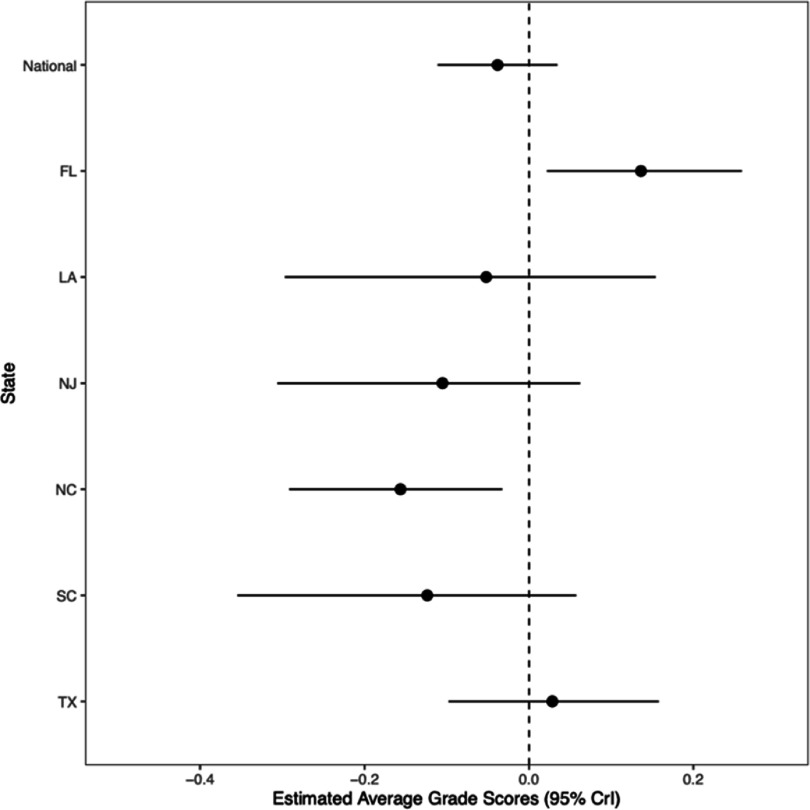
Estimated association between hurricane-force tropical cyclone exposure and 2009–2018 average standardized math grade scores. Dots indicate point estimates; whiskers, 95% credible intervals.

There was no association in the national model between hurricane-force tropical cyclone exposure and standardized math test scores (*β* = −0.04; 95% CrI: −0.11, 0.03; PP[*β* < 0] = 85%). State-specific results showed that counties in North Carolina exposed to hurricane-force tropical cyclones performed worse in math than non-exposed counties (*β* = −0.16; 95% CrI: −0.29, −0.03; PP[*β* < 0] = 99.4%) (figure 2, table S2). In contrast, counties in Florida exposed to hurricane-force tropical cyclones performed better in math than non-exposed counties (*β* = 0.14; 95% CrI: 0.02, 0.26; PP[*β* > 0] = 99.0%) (figure 2, table S2).

### 3.3. Association of hurricanes with RLAs scores

**Figure 3.**
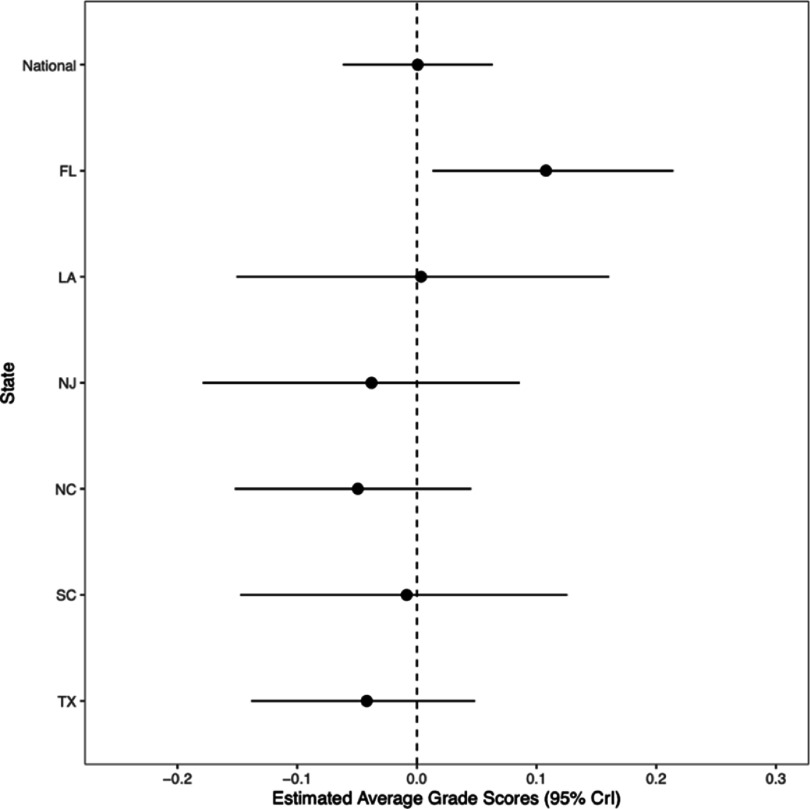
Estimated association between hurricane-force tropical cyclone exposure and 2009–2018 average standardized RLA grade scores. Dots indicate point estimates; whiskers, 95% credible intervals.

There was no association in the national model between hurricane-force tropical cyclone exposure and RLA scores (*β* = 0.00; 95% CrI: −0.06, 0.07; PP[*β* > 0] = 55.5%). State-specific results showed that counties in Florida exposed to hurricane-force tropical cyclones performed better in RLA than unexposed counties (*β* = 0.11; 95% CrI: 0.02, −0.04; PP[*β* < 0] = 99.9%) (figure 3, table S2).

### 3.4. Association of covariates with test scores

We observed several notable associations between grade cohort and county-level sociodemographic characteristics and average standardized test scores (figures S1 and S2). Grade cohorts with greater proportions of racialized and minoritized students (e.g. Black, Hispanic, Indigenous) tended to perform worse than average grade cohorts in both math and RLA. In contrast, those with greater proportions of students racialized as Asian tended to perform better than the national average cohort in both math and RLA. Grade cohorts with greater shares of students receiving free lunch tended to perform worse in math, but better in RLA. Grade cohorts with more socioeconomically disadvantaged students tended to perform worse than the national average grade cohort in only RLA (figures 2 and 3, supplemental table).

At the county level, counties with higher poverty levels tended to perform worse in math. Those with greater shares of English language learners tended to perform better than average in math, but worse in RLA. Counties with higher rates of college-educated adult residents tended to perform better than average in both math and RLA. In addition, counties with greater shares of urban schools and special education students tended to perform better than average in RLA (figures S1 and S2, table S2).

### 3.5. Sensitivity analysis

Most of the sensitivity analyses we conducted showed similar results to the main model with some exceptions. In the main, state-specific model excluding all covariates, we observed a negative association between counties exposed to hurricane-force tropical cyclone exposure and RLA scores in Texas (*β* = −0.11; 95% CrI: −0.22, −0.01; PP[*β* < 0] = 98%). In the state-specific model restricted to those counties that experienced only one hurricane in the study period excluding all covariates, we observed a positive association between hurricane exposure and RLA scores in counties in North Carolina (*β* = 0.20; 95% CrI: 0.09, 0.32; PP[*β* > 0] = 99.9%), and a negative association in counties in Texas (*β* = −0.17; 95% CrI: −0.26, −0.09; PP[*β* < 0] = 99.9%)

## 4. Discussion

In this comprehensive analysis of the association between hurricane-force tropical cyclones and educational attainment in the United States, we found that although hurricane-force tropical cyclones were not associated with standardized test performance in math or RLA on the national level, we observed associations for certain states. Accounting for both grade cohort- and county-level time varying characteristics, we found that hurricane-force tropical cyclones were associated with higher math and RLA scores in Florida and lower math scores in North Carolina.

There are several factors that may negatively influence a child’s long-term educational vulnerability during and following hurricanes. These include the destruction of school buildings and loss of vital records; prolonged school closure; displacement of students and teachers leading to delayed enrollment and multiple school changes; family separation and financial instability; unwelcoming and unsupportive new school environments following relocation; poor academic performance pre-disaster; the loss of a parent in the disaster; and increased work demands to compensate for lost income and assets (Peek 2008, Peek and Richardson 2010, Jahan *et al* 2022). All of these stressors could also compromise cognitive functioning and subsequent academic achievement (Pfefferbaum *et al* 2016).

There is evidence from the literature pointing to the negative consequences of hurricanes and other disasters on child education. Scott *et al* found that fourth to eighth grade New Orleans students exposed to Hurricane Katrina exhibited more aggressive behavior, and in turn, had worse academic achievement (Scott *et al* 2014). In this same cohort of students, Weems *et al* found that students exposed to the hurricane had greater posttraumatic stress, which predicted test anxiety, which was negatively associated with academic achievement (2013). Ward *et al* found that Mississippi students displaced by Katrina had both lower academic performance and were more likely to engage in negative behaviors, patterns that persisted two years following the storm (2008). On the school level, Holmes found that if the 1999–2000 storms in North Carolina had not occurred, twenty more schools throughout the state would have met their academic standards (Holmes 2002). Lai *et al* (2019) studied public schools affected by 2008 Hurricane Ike and found that attendance and rates of economically disadvantaged students were significant risk factors for worse academic recovery trajectories (Lai *et al* 2019). Although counties in Louisiana were exposed to hurricane-force tropical winds during our study period, we did not observe any significant effects of hurricane exposure on standardized test scores in that state.

For many states, we observed null associations between hurricane exposure and educational test scores, and in a few states, exposure appeared to increase test scores. These results may reflect state-level education policies that are implemented in the post-disaster context that influence their schools’ and students’ vulnerability or resilience. For example, Florida, a state that is prone to hurricanes, has policies in place such as make-up instructional days for schools, as well as resources available to support special education students (Anderson 2022, Solochek 2023). States such as North Carolina, on the other hand, may not have had the infrastructure in place to effectively withstand the deleterious effects of storms on their students’ academic achievement (Mack 2018, The Innovation Project 2019). Whereas states like Florida, where storms are more frequent, appear to have more easily accessible guidelines and an emphasis on returning students to school as quickly as possible, North Carolina’s return-to-school guidelines are less publicly accessible, and its schools seem slower to reopen post-disaster (Fuller and Davis 2021). It is also possible that states where we observed positive or null relationships between hurricanes and test scores received large influxes of federal disaster relief funding (U.S. Department of Education n.d.). Another possibility is that in these states, test scores only reflected the performance of more privileged students who were less impacted by the hurricane; more vulnerable, racially minoritized or socioeconomically disadvantaged students may be more likely to have been exposed to storm-related stressors and/or been displaced, not have been enrolled in or attended school, and therefore not have taken standardized tests (Picou and Marshall 2007, Fussell *et al* 2010, Peek and Richardson 2010, Bolin and Kurtz 2018). It is also possible that displaced students were relocated to schools with more resources and funding than their original ones, which may have mitigated potential negative effects on academic achievement (Pane *et al* 2008). As a result, we suggest that education policymakers facilitate the entry of displaced students into new school settings and provide supporting guidelines for affected schools like those readily available in Florida. There is a large body of literature pointing to voluntary migration in the aftermath of natural disasters, influenced by social, economic, and cultural factors (Hauer *et al* 2020, Chumky *et al* 2022, Sheldon and Zhan 2022). Our results may therefore be partially explained by the fact that post-disaster migration would have changed the sociodemographic composition of families in a hurricane-impacted county, thus indirectly affecting its students’ educational outcomes.

Our findings consistently demonstrated the educational vulnerability of racially and socioeconomically marginalized groups, regardless of hurricane exposure. Grade cohorts with greater shares of students racialized as Black, American Indian/Alaska Native, Hispanic, and who were socioeconomically disadvantaged performed more poorly on standardized testing in both math and RLAs. This comports with previous findings that these groups are at a systematic disadvantage in terms of standardized testing and overall educational attainment (White *et al* 2016, Gordon and Cui 2018) and further speaks to the need for post-storm resources to be targeted in this direction. In contrast, grade cohorts with greater shares of students racialized as Asian tended to perform better overall, which scholars attribute to unique cultural attributes (Hsin and Xie 2014, Liu and Xie 2016, Li and Xie 2020). Counties with greater shares of special education students tended to perform better on standardized testing, which may be indicative of the fact that the individual education programs (IEPs) required for this unique cohort may help schools identify and support this particularly vulnerable population. Indeed, the designation of a child with an IEP can be leveraged by schools to identify and prioritize students most at-risk post-hurricane and help to ensure they receive necessary supports. Counties with greater shares of English language learner students may have had worse RLAs scores due to language barriers (Bailey 2007). County-level socioeconomic status based on those living in poverty and residents with a college degree also tended to be strongly associated with academic performance.

This study has several limitations. First, standardized testing is not a complete representation of students’ academic success and potential as opposed to a more holistic measure such as grade point average or teacher-observed qualitative measures. However, unlike grade point average, which is weighted differently across schools, standardized test scores are easily accessible and comparable across school districts, counties, subjects, and time. Second, potentially salient covariates on the grade cohort and county levels were not available in the SEDA dataset, including grade cohort gender composition; county rates of public, private, and charter schools; or variables pertaining to school performance or funding. Third, the county was the smallest spatial unit available to capture hurricane exposure, test scores, and relevant covariates. Given the large size of counties and the many diverse schools and likely differential hurricane impacts within each of them, future analyses should consider using a more granular spatial unit of analysis such as a census tract or school district to have greater variance and better capture actual hurricane exposure and grade cohort composition. Fourth, if students had been displaced by hurricanes, their test scores would have been reflected in their new, rather than original, counties of residence and/or schooling. Fifth, our metric of hurricane exposure was solely based on wind speed, whereas hurricane impacts could perhaps have been more comprehensively captured by accounting for the extent of rainfall and/or structural damage. Sixth, we did not have any data available to us on school closures. Grouping together all counties that experienced high wind speeds irrespective of whether their schools closed may have attenuated the association of interest. Hurricane impacts on educational outcomes may have been far worse in those counties that experienced actual school closures in the aftermath of hurricanes during our study period. Lastly, we made two major assumptions in our analysis. We first assumed that the effect of hurricanes on test scores remained the same across time even when several years of recovery may have passed. We also assumed that hurricanes are rare events, but that certain states and counties are more frequently exposed to hurricanes than others. We only partially accounted for this by including random effects by state in a sensitivity analysis. Under the current trajectory of climate change, the rarity of hurricanes may not hold as they become more powerful and frequent. Greater intensity and potential regularity of hurricanes therefore warrants further investigation.

## 5. Conclusion

This study shows that educational outcomes associated with hurricane exposure are not only highly variable by state, but that disparities in academic performance persist across racial/ethnic and sociodemographic lines. To increase children’s educational resilience to the effects of hurricanes, policymakers should address both disaster-related educational procedures and policies, as well as underlying sociodemographic educational disparities by focusing on four key aspects of school recovery—emotional, academic, financial, and physical—detailed by the U.S. Government Accountability Office (Nowicki 2022). This will not only enhance student, but also community resilience to the effects of climate change-driven extreme weather events.

## Data Availability

The data used in this study were created from the following datasets: Tropical cyclone and hurricane exposure data are available as a package developed by Dr Anderson via https://github.com/geanders/hurricaneexposure and https://github.com/geanders/hurricaneexposuredata. County-level standardized test score and covariate data from 2008/2009–2017/2018 are available via the Stanford Education Data Archive Version 4.1: https://purl.stanford.edu/xv742vh9296. The data that support the findings of this study are openly available at the following URL/DOI: https://github.com/sparklabnyc/tropical_cyclones_educational_attainment_2022.

## Code availability

All code for analysis and visualization presented in this manuscript is available at https://github.com/sparklabnyc/tropical_cyclones_educational_attainment_2022.
